# Self-Shadowing Deposited Pure Metal Nanohelix Arrays and SERS Application

**DOI:** 10.1186/s11671-015-1205-8

**Published:** 2015-12-29

**Authors:** Yi-Jun Jen, San Chan, Jyong-Wei Huang, Ci-Yao Jheng, Wei-Chih Liu

**Affiliations:** Department of Electro-Optical Engineering, National Taipei University of Technology, 10608 No. 1, Sec. 3, Chung-Hsiao E. Rd, Taipei, Taiwan

**Keywords:** Glancing angle deposition, Nanohelix, SERS, g-factor

## Abstract

In this work, one-step glancing angle deposition is utilized to fabricate gold and silver nanohelix arrays (NHAs) on smooth glass substrates. During deposition, the substrate is cooled using liquid nitrogen and rotated with a tunable spin rate. The substrate spin rate is tuned to match the deposition rate to yield a spiral-like helix structure. The morphologies and optical properties of spiral-like Ag and Au NHAs are measured and compared. The polarization-dependent reflectance of Au NHA leads to a strong g-factor. The three-dimensional nanohelical structures are demonstrated to be a highly sensitive surface-enhanced Raman scattering (SERS) substrate.

## Background

Helical plasmonic structures have attracted considerable attention owing to their extraordinary optical properties, such as negative refraction [1], and strong circular dichroism; they can potentially be used in broadband circular polarizers [2] and biosensing [3]. The strong chirality is induced from a metal helix array that supports different plasmonic modes for left-handed circular polarized (LCP) and right-handed circular polarized (RCP) incident waves [4]. In 2009, a gold helix array was for the first time formed by direct laser writing [2]; it had a radius of curvature of approximately 0.7 μm and an arm width of around 1 μm. Such a gold helix array can act as a circular polarizer at terahertz frequencies. Unfortunately, it currently has insufficient resolution to make three-dimensional features that are smaller than 100 nm to provide chiroptical activity in the visible range. Recent progress has been made in reducing the feature size of plasmonic structures using glancing angle deposition (GLAD) [5], which is easy to implement and widely used to fabricate various nanostructured thin films [6–10].

The main advantage of GLAD is that it allows for one-step fabrication over a large area. Various metallic nanostructures can be grown on a smooth surface by the shadowing effect [11] in the initial stage of film growth. However, the main challenge in depositing metal nanostructures is that silver and gold adatoms have high mobilities, causing surface diffusion that precludes the self-shadowing effect. In some works, slanted silver nanorod arrays have been grown with extremely high deposition angles [12] from 86° to 89°, but more complicated three-dimensional structures remain difficult to grow. Recently, substrate cooling was conducted to reduce the surface mobilities of adatoms to enable the growth of a gold nanohelix array on a patterned substrate [6]. Such a gold helix array exhibits circular dichroism in the visible regime. However, the formation of a patterned substrate on whose surface seeds are regularly distributed requires a complicated and expensive lithographic procedure. One strategy to obliquely deposit three-dimensional plasmonic helices is to co-deposit two metals to form alloy plasmonic helices [13, 14]. The co-deposition changed the film growth state in the structure zone model to reach an equivalent condition corresponding to substrate cooling. Pure metal helices on a smooth surface without patterning are desired to be developed with a one-step procedure for deposition.

In this work, silver and gold nanohelix arrays (NHAs) are fabricated on non-seeded glass substrates with GLAD in a substrate cooling system. Self-shadowing deposition is achieved by ensuring the high directivity of the vapor flux. During deposition, the substrate is tilted with respect to the direction of incident flux and spins with a rate of *ω* that is optimized to the formation of spiral-like NHAs. Silver and gold spiral-like NHAs were successfully deposited on non-seeded glass substrates. At the corners of the metal NHAs were “hot spots” where the electric fields were stronger than the field along a straight metal nanorod. Additionally, the extension of a SERS substrate from a two-dimensional nanostructure to this three-dimensional nanostructure increases its overall surface area [15], enabling the adsorption and detection of more target molecules; therefore, a three-dimensional NHA is expected to exhibit a stronger SERS response than straight nanorod arrays. Here, the SERS from these NHAs are measured and compared with that of a silver nanorod array that has been demonstrated to be highly sensitive in the identification of viruses and bacteria.

## Methods

Nanohelices were deposited in an electron evaporation system; during this process, the substrate normal was tilted at an angle of 89° from the direction of incidence of the vapor. The center of the substrate and the evaporation source were vertically separated by 350 mm. Liquid nitrogen was passed through a loop under the substrate to cool the substrate holder to −140 °C. Pumping yielded a background pressure of 4 × 10^− 6^ Torr before evaporation. The deposition rate was maintained at 0.3 nm/s. The rate of rotation of the substrate was varied from 0.017 to 0.035 rpm to match the deposition rate and thereby optimize the helical nanostructure.

**Fig. 1:**
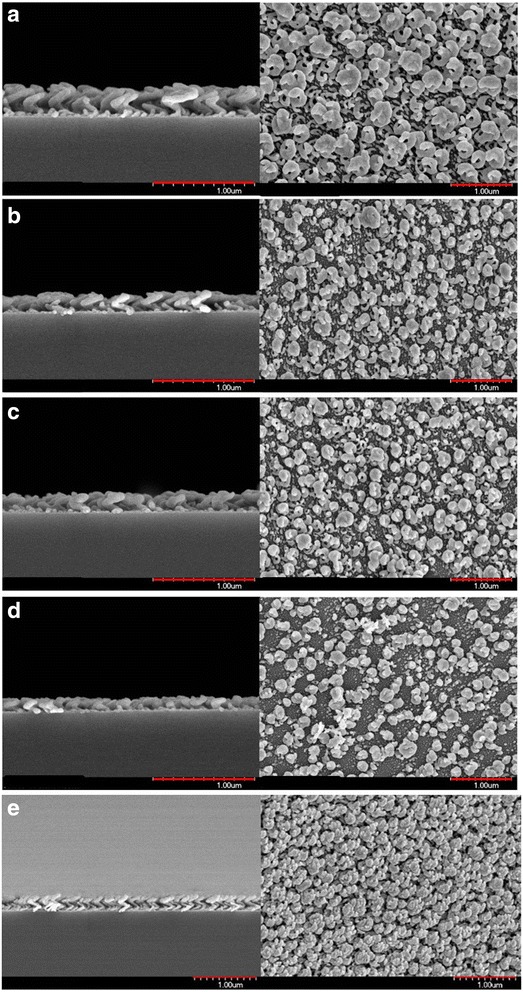
Top-view and cross-section SEM images of 1.5-turn Ag NHAs deposited at spin rates of **a** 0.017, **b** 0.023, **c** 0.029, and **d** 0.035 rpm and **e** 1.5-turn Au NHA deposited at a spin rate of 0.029 rpm

Figure 1a–d presents the top-view and cross-section scanning electron microscopic (SEM) images of 1.5-turn Ag NHAs that were deposited at spin rates *ω* of 0.017, 0.023, 0.029, and 0.035 rpm. A spiral-like Ag NHA was grown with *ω* = 0.017 rpm. Substrate spin rates of 0.023, 0.029, and 0.035 rpm yielded screw-like Ag NHAs. A higher spin rate of 0.035 rpm caused the Ag nanostructures to grow almost as an upright nanorod array. NHAs were grown on a smooth substrate under suitable deposition conditions. For Ag NHAs, the optimum growth conditions are the spin rate of *ω* = 0.017 rpm and deposition rate of 0.3 nm/s. The average diameter of the arms of the spiral-like Ag NHAs was 66 nm. The average pitch and radius of curvature were 153 and 88 nm, respectively. In the top-view SEM image in Fig. 1a, helices with pitch numbers from 0.5 to 1.5 are randomly distributed on the substrate surface.

For Au NHAs, the optimum growth conditions are the spin rate of *ω* = 0.029 rpm and deposition rate of 0.3 nm/s to have a spiral-like NHA. Figure 1e shows the cross-section and top-view SEM images of the 1.5-turn Au NHA. The average diameter of the arms was 58 nm. The average pitch and radius of curvature were 162 and 78 nm, respectively. Most of the gold helices had a pitch number of 1.5. The pitch angle, defined as the angle between the initial direction of growth of the rods and the substrate surface, was 29° for Au NHA and 27° for Ag NHA. The top views of the Ag and Au NHAs reveal that effects of competition during atomic self-shadowing growth are different between them. The surfaces of the substrates were populated randomly with nanohelices of different sizes and pitch numbers. The 1.5-pitch nanohelix densities of the two spiral-like NHAs were σ_Ag_ = 29 μm^− 2^ and σ_Au_ = 48 μm^− 2^. The fraction of complete nanohelices with a pitch number of 1.5 on the Au NHA was relatively high.

## Results and Discussion

**Fig. 2:**
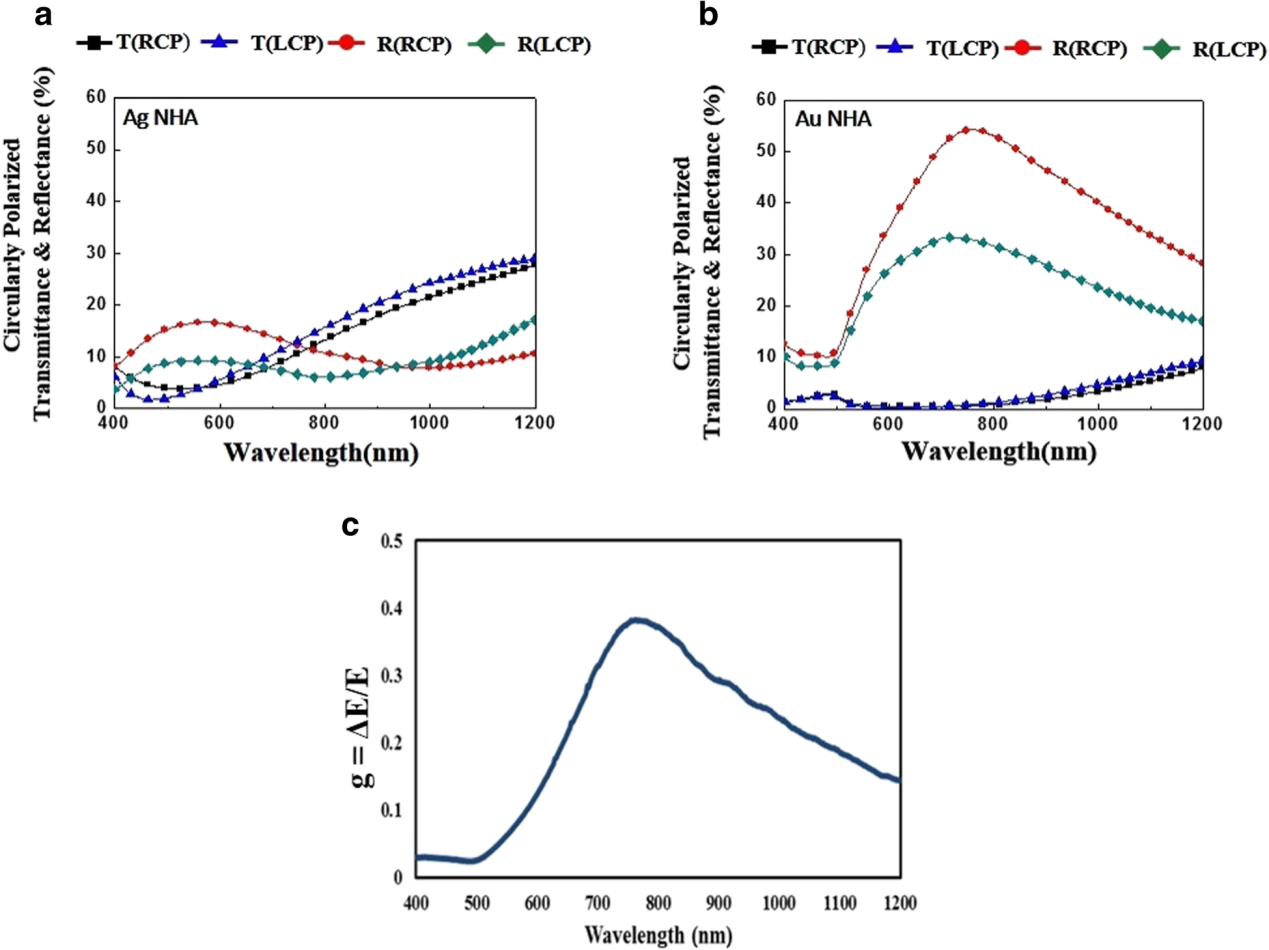
Circularly polarized transmittance and reflectance spectra of **a** Ag NHA and **b** Au NHA. **c** g-factor spectra of Au NHA

The transmittance and reflectance spectra of the two spiral-like NHAs deposited at *ω* = 0.017 rpm and *ω* = 0.029 rpm were measured with LCP incident light and RCP incident light, respectively, as shown in Fig. 2. Each LCP or RCP spectrum exhibits a transmittance minimum at a visible wavelength. For the Ag NHA, the LCP and RCP transmittance minima are 1.71 % at a wavelength of 471 nm (*λ* = 471 nm) and 3.8 % at a wavelength of 533 nm (*λ* = 533 nm), respectively. Both LCP and RCP transmittance spectra raise from their minima to approximately 28 % at *λ* = 1200 nm. The difference between LCP transmittance and RCP transmittance Δ*T* = *T*_LCP_ − *T*_RCP_ is below 3.13 % over wavelengths from 400 to 1200 nm. Both LCP and RCP reflectance spectra have localized maxima values of 9.3 % and 16.66 % at *λ* = 553 nm and *λ* = 575 nm, respectively. The maximum reflectance difference between LCP and RCP is 7.4 % at *λ* = 579 nm.

For the Au NHA, both LCP and RCP transmittance spectra are below 9.34 % and the transmittance difference Δ*T* = *T*_LCP_ − *T*_RCP_ is less than 1.73 % over the wavelengths from 400 to 1200 nm. However, the RCP and LCP reflectance spectra are higher than those of the Ag NHA. Both RCP and LCP reflectance spectra have peak values of 37.14 % and 25.54 % at *λ* = 755 nm and *λ* = 719 nm, respectively. The reflectance difference Δ*R* = *R*_RCP_ − *R*_LCP_ for Au NHA is significant: Δ*R* increases from −0.01 % at *λ* = 506 nm to the maximum value of 12.11 % at *λ* = 779 nm and then decays to 6.26 % at *λ* = 1200 nm.

Even though Au and Ag NHAs have similar pitch length and arm diameter, the polarization dependence of reflectance is significant for the Au NHA. Therefore, the g-factor is calculated for the Au NHA, as shown in Fig. 2c. The g-factor is the difference between the extinctance under LCP illumination and that under RCP illumination, Δ*E*, divided by the average extinctance *E*: *g* = *ΔE*/*E*. The g-factor of Au NHA remains around 0.03 from *λ* = 400 nm to *λ* = 510 nm, increases to 0.382 at *λ* = 763 nm, and then decreases to 0.144 at *λ* = 1200 nm. The strong g-factor of the Au NHA comes from the density of gold helices with a pitch number of 1.5 that exceeds that of the silver helices.

**Fig. 3:**
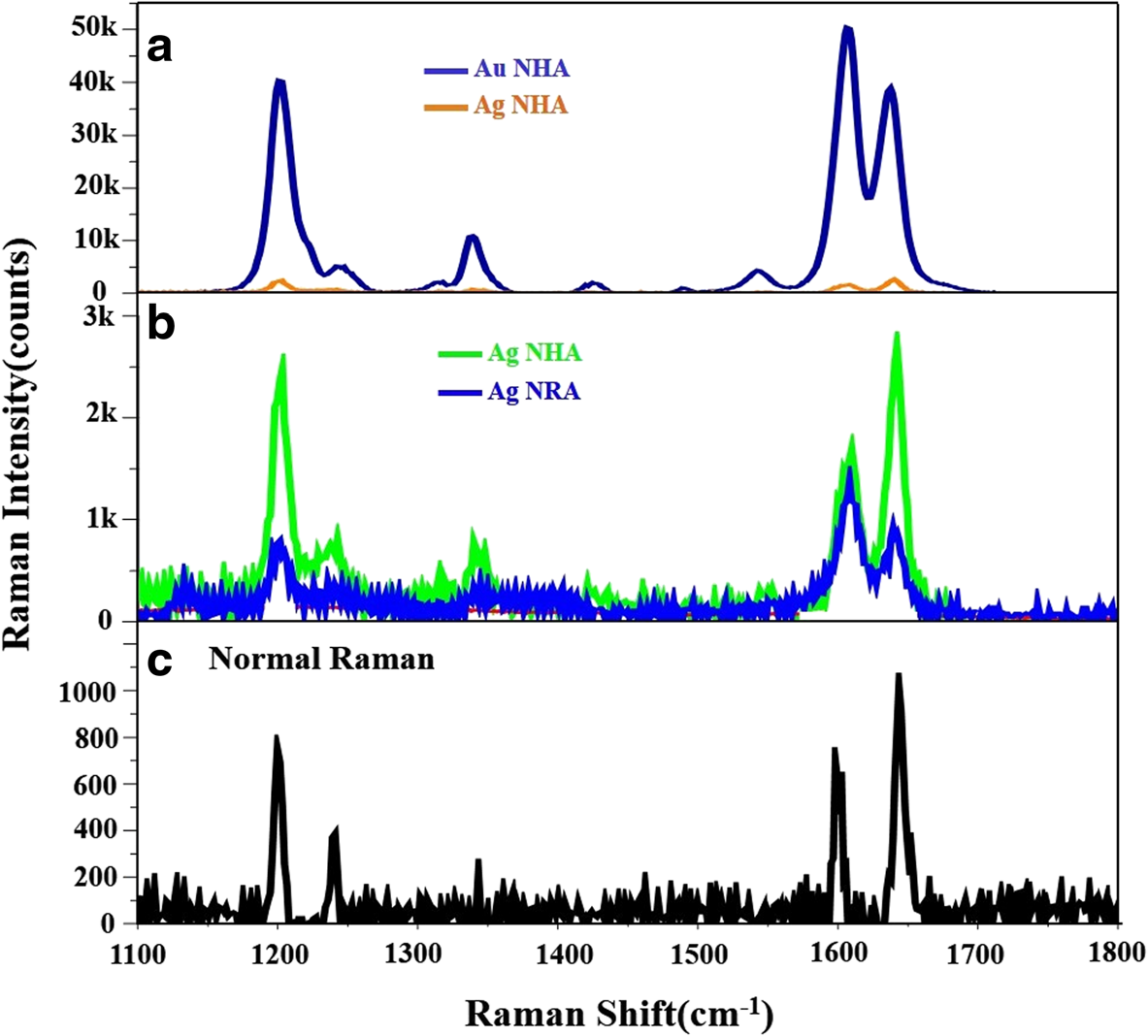
SERS spectra of **a** Au NHA and Ag NHA and **b** Ag NHA and Ag NRA. **c** Normal Raman spectra from 10^−2^ M BPE methanol solution

To perform SERS characterization, the Raman probe molecule, 1,2-di(4-pyridyl)ethylene (BPE, TCI), was used, and a 4-μL droplet of BPE methanol solution with a concentration of 5.5 × 10^−5^ M was dispersed on the surfaces of the three spiral-like metal NHAs. After the droplet had dried, the area over which it had spread on each of the substrates was observed to be approximately 5 mm^2^. The Raman spectra were obtained using a Stroker 785L Raman Spectrometer from Wasatch Photonics, with an excitation wavelength of 785 nm, a power of 100 mW, a laser spot with a diameter of less than 50 μm, and a collection time of 30 ms. The SERS spectra of the three metal NHAs were measured and compared with those of a slanted Ag nanorod array (NRA) that had been previously developed as a highly sensitive SERS substrate [16]. The Ag NRA was obliquely deposited by electron beam evaporation at a deposition angle of 89°. The average length of the Ag NRA was 211 nm. The normal Raman spectrum that was obtained from the BPE methanol bulk solution with a concentration of 10^− 2^M, and the SERS spectrum of the glancing-deposited Ag NRA were measured. The SERS intensity relative to the Raman intensity yielded an enhancement factor, EF [17], of around 10^4^, which matches a previously obtained result [16]. Figure 3 shows the experimentally obtained normal Raman spectrum and SERS spectra of BPE herein. All of the spectra include the following Raman characteristic peaks of BPE: Δ*ν* = 1200 cm^(−1)^ (C=C stretching mode) and Δ*ν* = 1610 cm^− 1^ (aromatic ring stretching mode) and Δ*ν* = 1640 cm^− 1^ (in-plane ring mode). At Δ*ν* = 1200 cm^(−1)^, the enhancement factors of the Ag NRA, Ag NHA, and Au NHA are ${\mathrm{EF}}_{\mathrm{Ag}\;\mathrm{N}\mathrm{H}\mathrm{A}}^{1200}$ = 3 × 10^4^, ${\mathrm{EF}}_{\mathrm{Ag}\;\mathrm{N}\mathrm{H}\mathrm{A}}^{1200}$ = 1 × 10^5^, and ${\mathrm{EF}}_{\mathrm{Au}\;\mathrm{N}\mathrm{H}\mathrm{A}}^{1200}$ = 1.8 × 10^6^, respectively. At Δ*v* = 1610 cm^− 1^, the enhancement factors of the three samples are ${\mathrm{EF}}_{\mathrm{Ag}\;\mathrm{N}\mathrm{R}\mathrm{A}}^{1610}$ = 3.6 × 10^4^, ${\mathrm{EF}}_{\mathrm{Ag}\;\mathrm{N}\mathrm{H}\mathrm{A}}^{1610}$ = 4.7 × 10^4^, and ${\mathrm{EF}}_{\mathrm{Au}\;\mathrm{N}\mathrm{H}\mathrm{A}}^{1610}$ = 1.4 × 10^6^. The intensities of the peaks of the Ag NHA are only approximately 2.9 times those of the Ag NRA. However, the intensities of the peaks of the Au NHA are at least 40 times higher than those of the Ag NRA. The Au NHA yielded the strongest SERS signal of the three NHAs samples. Since most of the Au nanohelices were developed with 1.5 turns, they were expected to exhibit many more hot spots than the other two samples.

## Conclusions

In conclusion, metal NHAs were successfully fabricated on a smooth substrate by GLAD. The substrate was cooled during deposition, and Ag and Au nanohelices were well formed using a deposition rate that matched the substrate spinning rate. Even under fixed deposition conditions, the morphologies of nanohelices varied with the metal used. A dense distribution of 1.5-pitch gold nanohelices exhibits strong circular dichroism and yields a SERS signal in the detection of an analyte with an ultra-low concentration. We envisage that the developed one-step self-shadowing deposition will provide a general route for the mass production of various metal nanohelices that involves the proper use of two-axis rotation stages in a cooling system. Such three-dimensional structured nanohelices have potential for biosensing applications.

## Abbreviations

GLAD: glancing angle deposition
LCP: left-handed circular polarized
NHAs: nanohelix arrays
NRA: nanorod array
RCP: right-handed circular polarized
SERS: surface-enhanced Raman scattering
